# Chemotherapy-induced CAF-associated stromal remodeling in a humanized stroma pancreatic ductal adenocarcinoma organoid xenograft model: a comparison between GP and GS

**DOI:** 10.1007/s13577-026-01443-8

**Published:** 2026-08-24

**Authors:** Kaoru Furihata, Waka Iwashita, Atsushi Kurabayashi, Mutsuo Furihata, Kazushige Uchida, Keisuke Taniuchi

**Affiliations:** 1 Department of Pathology, Kochi Medical School, Kochi University, Nankoku, Kochi 783-8505 Japan; 2 Department of Gastroenterology and Hepatology, Kochi Medical School, Kochi University, Kohasu, Oko-Cho, Nankoku, Kochi 783-8505 Japan

**Keywords:** Pancreatic ductal adenocarcinoma (PDAC), Cancer-associated fibroblast (CAF) subtypes, Organoid xenograft model, Preclinical drug evaluation, Stromal remodeling

## Abstract

Pancreatic ductal adenocarcinoma features a dense, desmoplastic tumor microenvironment (TME) dominated by cancer-associated fibroblasts (CAFs). While gemcitabine plus nab-paclitaxel (approximated as gemcitabine plus paclitaxel [GP]) and gemcitabine plus S-1 (GS) are standard regimens with distinct clinical indications, their specific effects on tumor suppression and CAF-associated stromal remodeling remain unclear. Using a humanized, three-dimensional (3D) S2-013 organoid xenograft model integrating human mesenchymal stem cells (MSCs) and human endothelial cells, we investigated the histopathological and immunohistochemical alterations induced by GP and GS. We quantitatively evaluated tumor parenchymal growth using cytokeratin 19 (CK19) and CAF-associated marker profiles, including α-smooth muscle actin (αSMA), fibroblast activation protein (FAP), platelet-derived growth factor receptors (PDGFR), and interleukin-6 (IL-6). Both regimens clearly suppressed tumor growth, but only GP significantly reduced parenchymal tumor growth compared with untreated controls. Notably, the treatments selectively remodeled the peritumoral stroma in the humanized xenograft model: PDGFR-expressing CAF-like cells were significantly reduced in the GP group, whereas FAP-expressing CAF-like cells and IL-6-expressing stromal cells were significantly decreased in the GS group. αSMA expression showed a general decreasing trend in both therapeutic arms. In summary, GP and GS chemotherapies induced distinct, measurable humanized TME remodeling modalities beyond direct cytotoxicity. By successfully recapitulating drug–stroma interactions and suggesting the human MSC origin of the peritumoral CAFs, this study demonstrates the robustness of our organoid xenograft model as a powerful preclinical screening platform for evaluating and predicting the efficacy of novel stroma-targeted and immunomodulatory investigative compounds.

## Introduction

The recent implementation of neoadjuvant chemotherapy has enabled tumor downstaging and increased the feasibility of secondary curative resection, particularly in patients with borderline resectable or locally advanced pancreatic ductal adenocarcinoma (PDAC). Thus, 30–40% of such patients can now undergo successful resection following neoadjuvant therapy [1, 2]. Among available regimens, gemcitabine plus S-1 (GS) has demonstrated survival advantages as a preoperative (neoadjuvant) therapy in resectable PDAC [3, 4], while gemcitabine plus nab-paclitaxel is an established first-line therapy for advanced or unresectable disease due to its superior outcomes compared with gemcitabine monotherapy [5].

PDAC is histologically characterized by a prominent desmoplastic stroma, with a tumor microenvironment (TME) featuring abundant cancer-associated fibroblasts (CAFs). Initially regarded as a homogeneous population responsible for extracellular matrix deposition, CAFs are now recognized as functionally heterogeneous, exhibiting subtype-specific roles in tumor biology [6, 7], and are broadly categorized into myofibroblastic CAFs (myCAFs)—located adjacent to tumor cells and highly express α-smooth muscle actin (αSMA), contributing to extracellular matrix (ECM) remodeling and fibrosis [8, 9]—and inflammatory CAFs (iCAFs)—located more distally from tumor cells, have low αSMA expression, and secrete inflammatory cytokines such as interleukin-6 (IL-6), promoting tumor progression and immune modulation [10].

These diverse CAF populations express distinct markers, including fibroblast activation protein (FAP), a membrane-bound serine protease that promotes ECM remodeling and is associated with tumor growth, invasion, and metastasis [11, 12], and platelet-derived growth factor receptors (PDGFRα/β), which are tyrosine kinase receptors expressed on fibroblasts and CAFs that regulate fibroblast proliferation and have been implicated in poor prognosis in various cancers, including PDAC [13–15].

Despite increasing evidence of CAF heterogeneity and their diverse roles in tumor progression, the efficacy of standard chemotherapeutic regimens on PDAC-stroma-associated tumor growth has not been established, particularly regarding their effects on CAF populations and heterogeneity in vivo. Furthermore, conventional single-cell-line xenografts notoriously fail to recapitulate the dense, active human stroma, limiting their utility in accurately screening next-generation stroma-targeted agents during preclinical phases [16]. Therefore, establishing a humanized tumor microenvironment (TME) that enables rigorous assessment of therapy-induced stromal dynamics is highly desirable for translational drug evaluation [17]. In this context, we evaluated the histological impact of GS and gemcitabine plus paclitaxel (GP; used instead of nab-paclitaxel due to concerns about allergic reactions to human albumin in mice) on tumor growth and CAF-associated stromal marker expression, in a PDAC xenograft model established from human pancreatic cancer cell line (S2-013)-derived organoids. The S2-013 organoid xenograft we established faithfully recapitulates human PDAC histology and architecture, demonstrating translational utility for regimen testing [18]. Notably, in a previous S2-013 organoid xenograft study by our group, the combination of rapamycin with GP synergistically inhibited tumor growth, demonstrating the suitability of this model for evaluating clinically relevant drug combinations [19]. We further explored whether the model could recapitulate the relative efficacy observed in clinical settings. Based on our previous findings, we investigated the potential therapeutic efficacy of GP and GS treatments using a reconstituted, humanized stroma xenograft model established from S2-013 organoids co-cultured with human MSCs and human umbilical vein endothelial cells (HUVECs). Using histopathological and immunohistochemical techniques, we assessed tumor response and CAF behavior by measuring the expression levels of αSMA, FAP, PDGFR, and IL-6. This investigation would clarify the specific changes in stromal remodeling and CAF subtype activation induced by these treatment regimens in PDAC xenografts, thereby providing a robust platform for future preclinical drug evaluation.

## Materials and methods

### Cell culture and organoid preparation

The human PDAC cell line S2-013, a subline of SUIT-2, was obtained from the Cell Resource Center for Biomedical Research, Institute of Development, Aging and Cancer, Tohoku University (Sendai, Japan). S2-013 cells were cultured in Dulbecco’s modified Eagle’s medium (DMEM; Fujifilm Wako Pure Chemical Corporation, Osaka, Japan) supplemented with 10% fetal bovine serum and maintained at 37 °C in a humidified incubator. HUVECs and human mesenchymal stem cells (MSCs) were expanded under standard culture conditions as described previously [18]. All cell lines were confirmed mycoplasma-free using the TaKaRa PCR Mycoplasma Detection Set (Takara Bio Inc., Kusatsu, Japan) in our laboratory prior to the experiments. Organoids derived from the S2-013 cell line were generated by co-culturing S2-013 cells with HUVECs and MSCs, as described in established protocols [18]. To establish xenograft formation, a single S2-013-derived organoid suspended in 50 μL Matrigel/DMEM mixture was implanted subcutaneously into the flank region of 7-week-old female athymic nude mice (BALB/cSlc-nu/nu; Japan SLC, Shizuoka, Japan). After xenograft implantation, tumors were allowed to develop for approximately 2 weeks before therapeutic interventions were initiated.

### Drug administration

Following ethical validation by the Institutional Animal Care and Use Committee of Kochi University (approval #S-00015), 7-week-old, specific-pathogen-free female athymic nude mice (BALB/cSlc-nu/nu) were obtained from Japan SLC, Inc. (Shizuoka, Japan) and raised in strict compliance with institutional guidelines. Therapeutic interventions were initiated 2 weeks after organoid implantation in all mice, using drug administration protocols modeled after the regimens previously established by Jobu et al. [19], with study-specific modifications optimized for the current investigation.

### GP regimen

The GP regimen consisted of intraperitoneal administration of gemcitabine (50 mg/kg) combined with paclitaxel (5 mg/kg) on days 1, 8, and 15 of each 28-day cycle. This schedule was administered for 6 weeks.

### GS regimen

For the GS regimen, gemcitabine (50 mg/kg) was administered intraperitoneally once weekly on days 1, 8, 15, and 22, followed by a 2-week rest period. S-1 was administered orally 5 days per week for 28 days according to the dosing schedule described previously [19], followed by a 2-week rest period. The regimen was then administered for another 2 weeks, for a total of 8 weeks.

### Tumor monitoring and tissue collection

Tumor dimensions were measured weekly using digital calipers. Volumes were estimated using the following formula:

V = 0.5 × (long axis) × (short axis)^2^,

where the long axis denotes the maximal tumor dimension, and the short axis denotes its orthogonal width. Following the humane endpoints approved by the Institutional Animal Care and Use Committee of Kochi University, all animals were monitored daily. Euthanasia was performed if mice exhibited severe weight loss (> 20%), ulceration, impaired mobility, or other signs of severe distress. All animals were managed strictly within these approved criteria throughout the study period. Mice were euthanized 1 week after the final treatment, and tumors, together with major organs, were collected for downstream histopathological analyses.

### Antibodies used

Anti-cytokeratin 19 (CK19) antibody (BA17) was purchased from Thermo Fisher Scientific (Waltham, MA, USA) and used as an epithelial marker for the S2-013 cell line. Anti-αSMA (Clone 1A4) antibody was purchased from Dako Cytomation (Glostrup, Denmark), while anti-IL-6 (1.2-2B11-2G10, ab9324), anti–PDGFRα+β (Y92, C-terminal, ab32570), and anti-FAP (EPR20021, ab207178) antibodies were obtained from Abcam (Cambridge, UK) and used as CAF-related markers. Anti-CD34 antibody (M7165) was also purchased from Dako Cytomation and used as an endothelial cell marker.

### Tissue processing and histology

Excised tissue specimens were fixed in 10% neutral-buffered formalin at ambient temperature for 24–48 h, dehydrated via a graded ethanol series, and embedded in paraffin wax blocks. For structural histopathological examination, 4-µm-thick sections were sliced and processed for routine hematoxylin and eosin (HE) staining.

### Evaluation of tumor growth in PDAC xenograft via histological and immunohistochemical analysis

To accurately evaluate tumor growth histologically, representative parenchymal lesions in PDAC xenografts were measured by CK19 immunostaining. Because immunoreaction with the CK19 antibody is restricted to the parenchymal epithelial components of PDAC xenograft, excluding necrotic or bleeding parenchymal parts and fibrous stroma, this measurement provides an accurate assessment of the precise rate of tumor diminution by GP and GS. CK19-positive areas were microscopically marked and quantified via manual tracing using the built-in area-calculation function of the Olympus D23 imaging system (Olympus Corporation, Tokyo, Japan). For each tumor, the area of the maximal cross-sectional plane (largest cut surface) was measured on the corresponding CK19-immunostained section. The total parenchymal area was calculated by summing all CK19-positive regions, excluding the stroma, as well as necrotic and hemorrhagic areas. All measurements were performed blinded to treatment allocation.

### Region of interest, field selection, and CAF scoring

CAF evaluation focused on the peritumoral stroma immediately adjacent to the tumor parenchyma, avoiding areas of overt necrosis or hemorrhage and distal stromal regions. All tumors from the untreated control group (*n* = 8), GP group (*n* = 6), and GS group (*n* = 8) were included in the CAF analysis. For each case, five non-overlapping high-power fields (HPFs; 200× magnification) were selected systematically using a grid approach. Observers were blinded to treatment allocation, with two pathologists independently scoring all slides.

For each HPF and each marker (αSMA, FAP, PDGFR, IL-6), CAF staining in spindle-shaped fibroblast-like stromal cells was scored as follows:

- ・Staining intensity (0–3): 0 = negative; 1 = weak; 2 = moderate; and 3 = strong.
- ・Positive cell proportion (0–4): 0 = 0%; 1 = 1%–10%; 2 = 11%–50%; 3 = 51%–80%; and 4 = 81%–100%.

The field score was the sum of the staining intensity and positive proportion (range 0–7). For each tumor, the case score was the mean of the five HPFs. Prespecified categories were applied to determine the staining grade: Grade 1 (low): total 0–2; Grade 2 (intermediate): total 3–5; Grade 3 (high): total 6–7.

### Statistical analysis

All analyses were performed using EZR (R-based GUI; Jichi Medical University Saitama Medical Center). For longitudinal tumor volume analysis, tumor growth over time was evaluated using a linear mixed-effects model with treatment group, time (weeks), and their interaction as fixed effects and a random intercept for each mouse. Statistical significance was assessed based on the treatment-by-time interaction. Between-group comparisons of quantitative histological data were performed using two-sided unpaired Student’s t tests, whereas comparisons of immunohistochemical staining grades were performed using the Mann–Whitney U test. A two-sided *p* < 0.05 was considered statistically significant.

## Results

### Evaluation of tumor suppression of GP and GS regimens on xenograft PDAC growth in the S2-013 organoid model

**Fig. 1:**
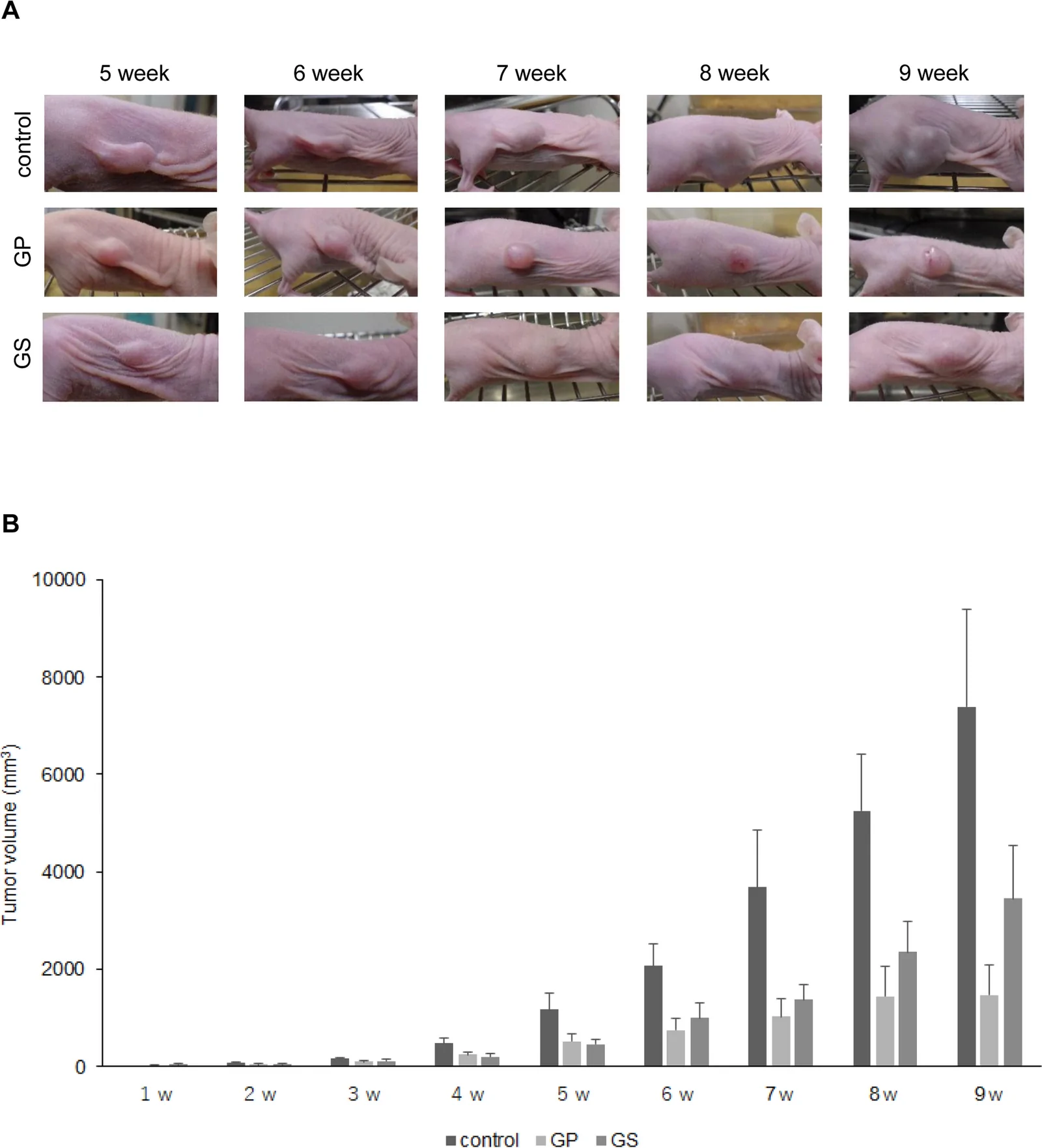
Macroscopic tumor growth and suppressive effects of gemcitabine plus paclitaxel (GP) and gemcitabine plus S-1 (GS) treatment regimens. **A** Representative images of pancreatic ductal adenocarcinoma (PDAC) xenograft derived from S2-013 organoids following subcutaneous transplantation in the treatment and control groups. Representative images of PDAC xenograft in mice at 5–9 weeks after subcutaneous transplantation of S2-013 organoids. Upper panels show the untreated control group; middle panels depict the GP group; and lower panels present the GS group. Tumor growth was monitored longitudinally throughout the observation period. **B** Tumor growth in PDAC xenografts of the treatment and control groups. Mice bearing S2-013 organoid-derived xenografts were first treated 2 weeks after transplantation. The untreated control group (*n* = 8), GP group (*n* = 6), and GS group (*n* = 8) were monitored weekly for tumor growth. GP treatment was administered for 6 weeks, whereas GS treatment was administered for a total of 8 weeks. Tumor growth over time was analyzed using a linear mixed-effects model. Significant treatment-by-time interactions were observed for both the GP and GS groups compared with the untreated control group (both *p* < 0.05). Bars represent mean tumor volume, and error bars indicate SEM

Two weeks after implantation of S2-013-derived organoids, the untreated control, GP, and GS groups received the first treatment, and tumor growth was macroscopically evaluated. The mean tumor volume, measured from the external surface of mice, increased in each treatment group. Tumor growth over time was analyzed using a linear mixed-effects model. Compared with the untreated control group, significant treatment-by-time interactions were observed for both the GP (*p* = 7.78 × 10⁻⁹) and GS (*p* = 2.02 × 10⁻⁶) groups, indicating significantly reduced tumor growth rates (Fig. 1A, B).

**Fig. 2:**
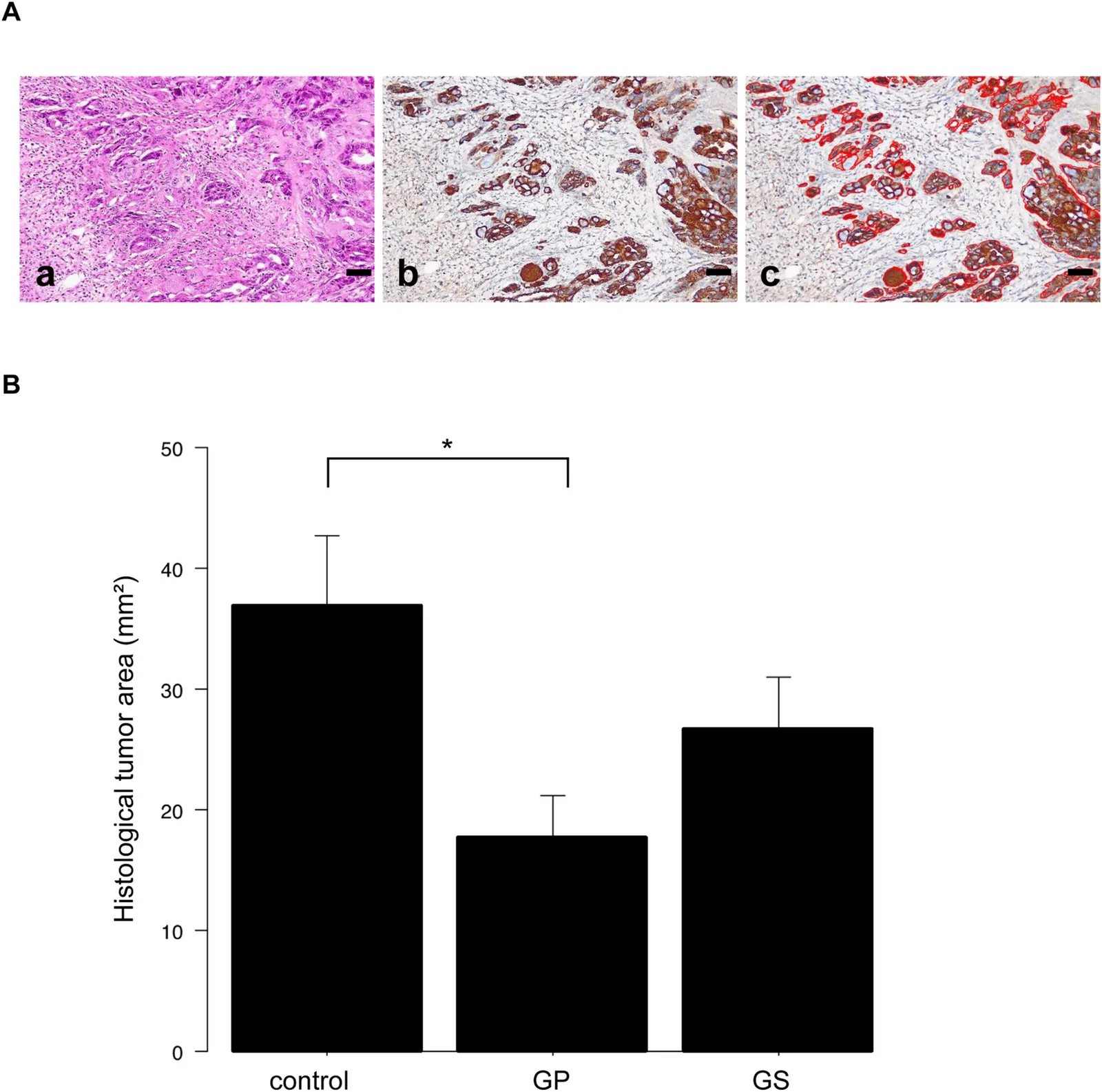
Histological and immunohistochemical quantification of parenchymal tumor growth. **A** Evaluation of tumor growth in pancreatic ductal adenocarcinoma (PDAC) xenografts via histological and immunohistochemical analyses. **a** Representative histological images of xenograft tumors stained with hematoxylin and eosin (H&E), showing parenchymal glandular duct formation with stromal fibrosis, recapitulating typical human PDAC histology. **b, c** Two consecutive sections were treated with a CK19 antibody to identify parenchymal epithelial components. To measure the representative parenchymal lesions of PDAC xenografts in each case, CK19-positive areas were microscopically delineated and selected for semi-automated quantification. Scale bars, 50 µm. **B** Antitumor effects on PDAC xenograft in each treatment and control group. Based on the quantified CK19-positive areas in each tumor, relative parenchymal size was compared among the control, GP, and GS groups. *p < 0.05, Student’s t test. Bars represent mean histological tumor area (mm^2^), and error bars indicate SEM. Parenchymal size significantly decreased in the GP group compared with the untreated control group

As shown in Fig. 2A, the histology of the xenograft tumors in the S2-013 organoid model predominantly featured parenchymal glandular duct formation with extensive stromal fibrosis, as commonly demonstrated in human PDAC histology. Vascular and perineural invasions were detected at low frequencies across all groups. One control case with regional lymph-node metastasis and two controls (one GP, one GS) with lung metastasis were found. Focal liver necrosis was observed in one case in the GP group, but no liver, bone marrow, or renal metastasis was detected in any group.

To quantify tumor volume microscopically, CK19-positive areas were determined in each tumor. Notably, the total CK19-positive parenchyma was significantly suppressed only in the GP group compared with the untreated group (Fig. 2B).

### Histopathological and immunohistochemical identification of CAFs among PDAC xenografts

**Fig. 3:**
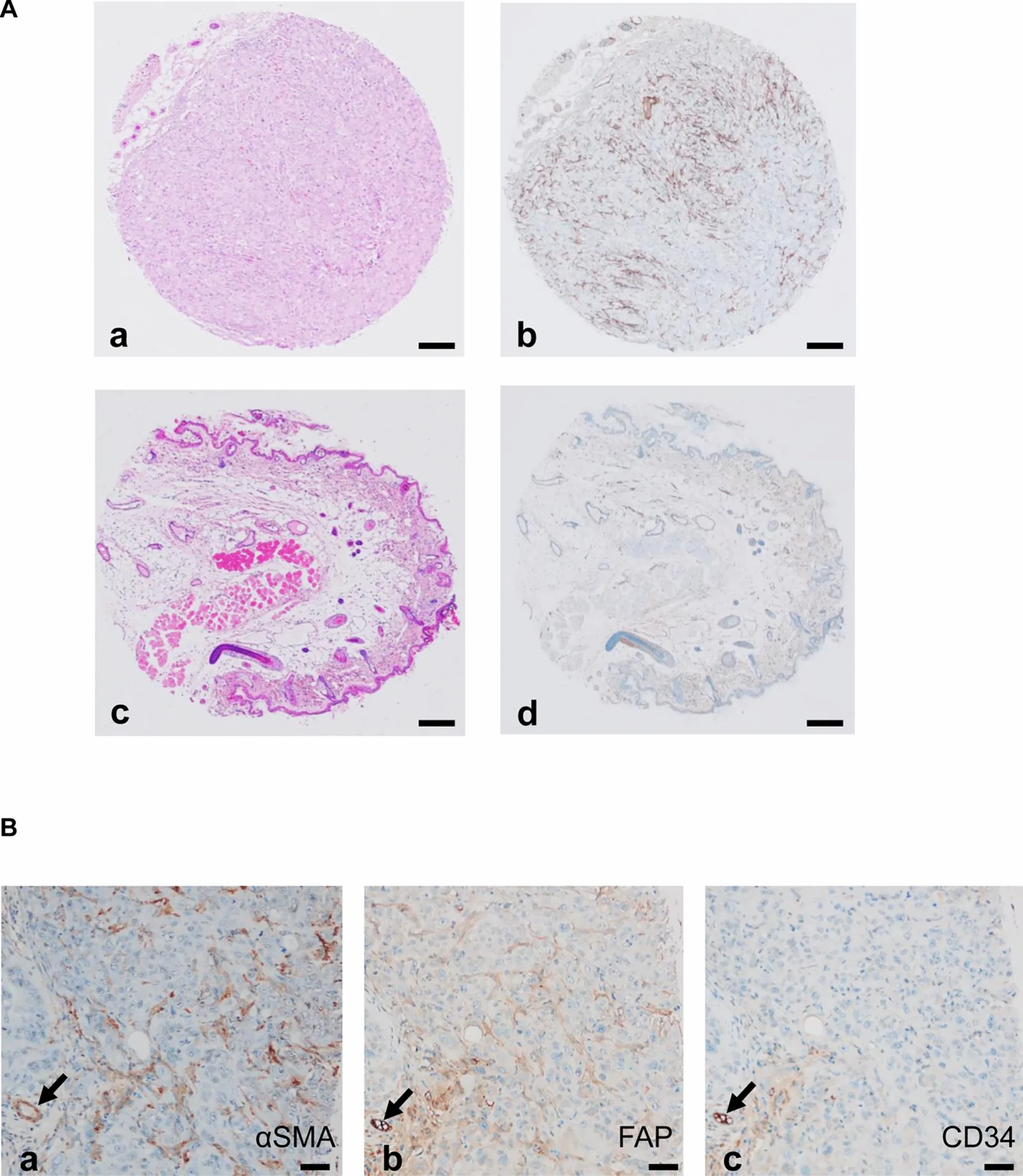
Identification and morphological characterization of cancer-associated fibroblasts (CAFs). **A** Comparison of α-smooth muscle actin (αSMA) staining between pancreatic ductal adenocarcinoma (PDAC) xenograft and murine control tissue. Two consecutive sections of xenograft tumor (**a** hematoxylin and eosin [H&E]; **b** immunoreaction with αSMA antibody), and murine control tissue (**c** H&E; **d** immunoreaction with αSMA antibody), respectively. Representative staining images with αSMA antibody. Strong and extensive αSMA-positive cells were detected in tumor areas (**b**), whereas only weak staining was observed in adjacent normal murine tissue (**d**). Scale bars, 500 µm. **B** Representative immunohistochemical staining of CAFs in PDAC xenograft. a, b, c: Three consecutive sections of PDAC xenograft presenting representative staining images of CAF markers in tumor tissue. Irregular, spindle-shaped cells surrounding the tumor cells were positive for αSMA (**a**) and FAP (**b**), whereas no CD34 staining was observed in these cells. Each arrow indicates positive controls in smooth muscle of a blood vessel (**a**) and endothelial cells (**b, c**). Scale bars, 50 µm

CAFs are spindle-shaped and exhibit positive αSMA immunoreactivity [20]. In the present study, stronger, more extensive αSMA-positive fibroblastic cells were immunohistochemically detected in the stromal areas surrounding the organoid-derived tumor glands than compared with adjacent non-tumorous control murine connective tissue. In contrast, such stromal cells were sparse in non-neoplastic murine tissue (Fig. 3A). These αSMA-positive fibroblastic cells also had a subgroup exhibiting FAP positivity. However, these cells were negative for CD34 by immunostaining (Fig. 3B). Based on these findings, we diagnosed the αSMA-positive fibroblastic cells as CAFs. Detected CAFs were predominantly localized among the tumor parenchyma and stroma closely adjacent to the parenchymal regions of the PDAC xenografts, rather than in stromal areas distant from or peripheral to the tumor. CAF distribution patterns did not differ significantly between PDAC areas adjacent to necrotic sites and other PDAC regions.

### Comparison of the distribution densities of the parenchymal CAFs between the GP and GS treatment groups and control groups

**Fig. 4:**
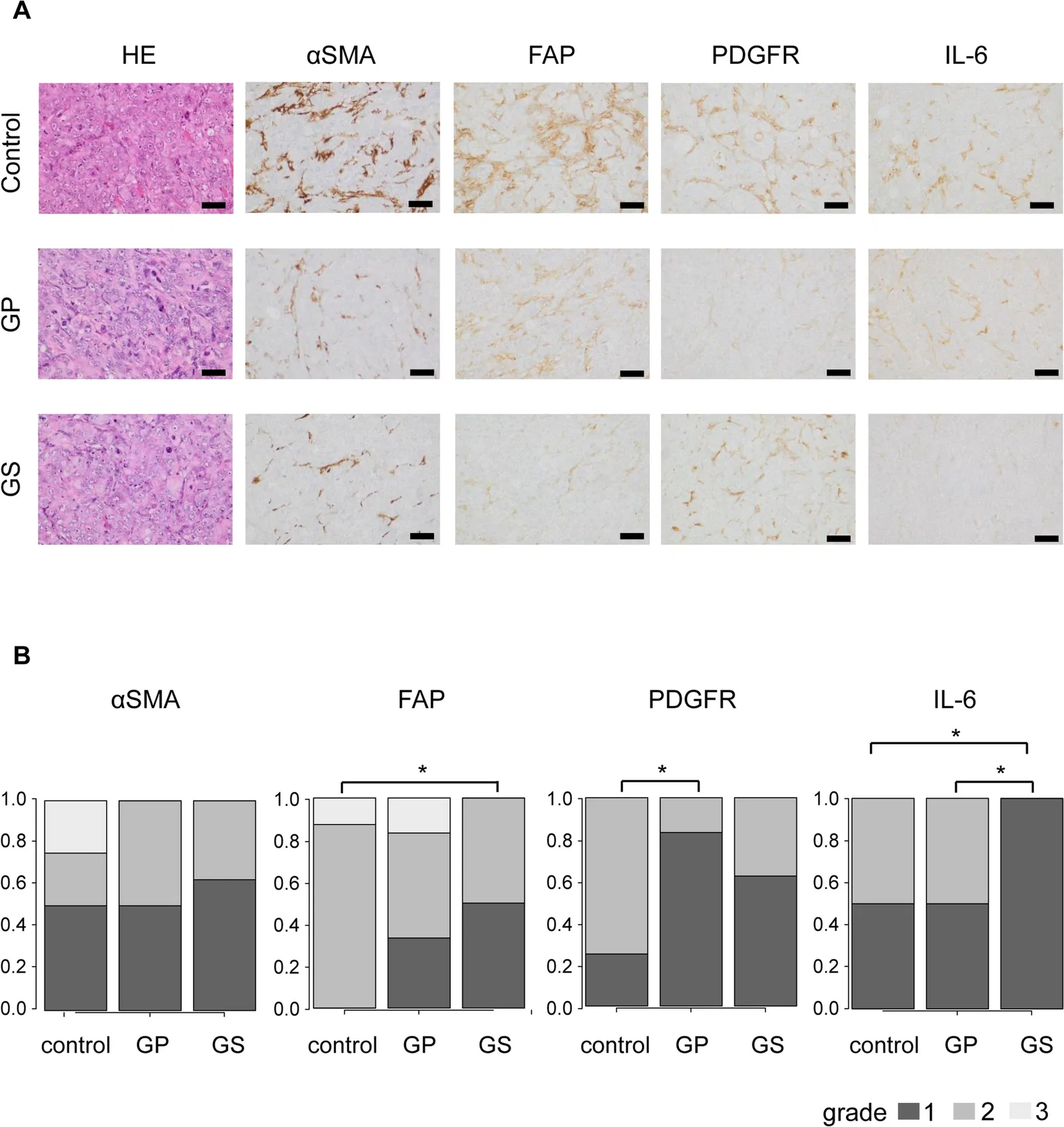
Differential impact of gemcitabine plus paclitaxel (GP) and gemcitabine plus S-1 (GS) chemotherapies on cancer-associated fibroblasts (CAF) marker expression and staining grades. **A** Representative immunohistochemical staining images of PDAC xenograft in each treatment group. Representative images were carefully selected to clearly demonstrate hematoxylin counterstaining, cell morphology, and correspondence with the semi-quantitative results. Representative immunohistochemical images of CAF marker expression in the control (upper row), GP (middle row), and GS (lower row) groups. From left to right: hematoxylin and eosin and immunohistochemical staining of αSMA, FAP, PDGFR, and IL-6. Scale bars, 50 µm. **B** Comparison of CAF marker staining grades in each treatment and control group. Based on the immunohistochemical findings shown in (A), the proportions of staining grades (1–3) for αSMA, FAP, PDGFR, and IL-6 were compared among the control (*n* = 8), GP (*n* = 6), and GS (*n* = 8) groups. No significant differences in αSMA staining grade were observed among the groups. However, the FAP staining grade of the GS group, the PDGFR staining grade of the GP group, and the IL-6 staining grade of the GS group significantly decreased. *p < 0.05, Mann–Whitney U test. Data are presented as cumulative bar charts showing the composition of each staining grade

Since the peritumoral stroma is a central component of TME and directly interacts with tumor cells, thereby contributing to tumor progression and drug response, we focused on this region to evaluate the distribution and density of CAF infiltration in PDAC xenografts using immunohistochemistry with CAF-related markers (αSMA, FAP, PDGFR, and IL-6). Representative immunohistochemical images of CAF markers (αSMA, FAP, and PDGFR) and their corresponding average staining intensities are summarized in Fig. 4. The distribution densities of αSMA-positive CAFs tended to be lower in the drug-treated groups (GP, GS) compared with the untreated group (control), with no significant differences. FAP distribution density was significantly lower in the GS group than in the untreated group, while PDGFR density was significantly lower in the GP group than in the untreated group. IL-6 distribution density was significantly lower in the GS group than in the other groups. Notably, FAP expression was virtually undetectable in all lung metastasis specimens derived from human PDAC organoid xenografts across all groups.

## Discussion

Recent advances in chemotherapy have improved outcomes for both resectable and unresectable PDAC. In this study, we used a human PDAC organoid transplant model to investigate the effects of two chemotherapy regimens (GP and GS) on tumor progression by altering the TME, as assessed histologically. However, we used GP rather than gemcitabine plus nab-paclitaxel. This was because nab-paclitaxel is formulated with human albumin, which can induce allergic and immunologic reactions in mice, thereby limiting its applicability in xenograft models. A previous study using the same S2-013 organoid xenograft model also adopted GP for the same reason, successfully demonstrating its utility in evaluating combination regimens [19]. Therefore, GP is a valid experimental alternative to gemcitabine plus nab-paclitaxel in this preclinical setting.

This PDAC organoid model reproduced the typical morphological and architectural characteristics of human PDAC tissue, including glandular duct formation, localized necrosis and hemorrhage, stromal fibrosis, and, in some cases, vascular and nerve tumor invasion.

The two treatment regimens effectively shrank tumors compared with the control group, especially GP, which significantly inhibited tumor growth. This superiority of GP over GS is consistent with clinical evidence, with GP demonstrating greater therapeutic efficacy, thereby supporting the translational relevance of this organoid xenograft model to human PDAC.

However, these findings do not necessarily imply that GP should universally replace GS in clinical practice. In human PDAC treatment, regimen selection is influenced not only by antitumor efficacy but also by treatment tolerability, patient performance status, surgical resectability, and the therapeutic setting, including neoadjuvant versus palliative intent. GS has been widely adopted as a neoadjuvant treatment for resectable PDAC because of its favorable balance between efficacy and tolerability, whereas GP is often preferred for advanced or unresectable disease requiring more intensive tumor reduction.

Only a limited number of pulmonary metastasis cases were identified in each group, including the control group, without significant differences, suggesting that both chemotherapies exerted their antitumor effects primarily by suppressing primary tumor growth rather than by inhibiting metastatic properties.

Interestingly, our findings further indicate that these regimens may exert distinct biological effects on the TME beyond direct cytotoxicity. GP predominantly reduced PDGFR-positive CAF-like stromal cells and was associated with greater tumor shrinkage, whereas GS preferentially suppressed FAP-positive and IL-6-expressing CAF-like populations. These differences suggest that GS may provide unique stromal or immunomodulatory benefits despite its relatively weaker tumor-reductive effect. Therefore, the therapeutic value of GS may not be fully reflected by tumor volume reduction alone, and the current findings signify that GP and GS may induce distinct stromal remodeling patterns beyond their direct antitumor effects.

Crucially, our co-culture organoid formulation directly incorporates human MSCs alongside S2-013 cells and HUVECs. In our model, robust and extensive αSMA and FAP immunoreactivity occurred exclusively within the peritumoral stroma, whereas the adjacent host murine connective tissue exhibited only sparse, negligible background staining. Although definitive host-versus-graft lineage tracing was not employed, this spatial dichotomy suggests that the detected CAFs may originate from embedded human MSCs transformed by malignant stromal cross-talk, rather than representing a nonspecific host murine reaction. Consequently, this model provides a potentially physiologically relevant, humanized stromal proxy for in vivo studies.

The aggressive nature of PDAC is mainly driven by the fibrous desmoplastic stroma [21]. CAFs, with major subtypes myCAFs and iCAFs [10], are significant fibrous components of the TME in PDAC and contribute meaningfully to tumor progression, immune evasion, and therapeutic resistance [6, 22]. Although iCAF populations are typically localized in more distal stromal regions, a subset of CAFs within the peritumoral stroma can exhibit iCAF-like characteristics, including low αSMA and high IL-6 expression [10]. Therefore, the observed changes in IL-6 and FAP expression in this study may partially reflect the dynamics of such CAF-like cells within the peritumoral stroma. Thus, our findings provide insights into the overall behavior of CAFs in the peritumoral stroma, potentially including both myCAF- and iCAF-like phenotypes.

FAP, a common indicator of CAF activation, is expressed in both myCAF and iCAF subtypes [22]. It is particularly abundantly expressed in CAFs, which not only promote tumor growth but also contribute to substrate remodeling and immunosuppression during TME establishment [11, 12, 23, 24]. Thus, FAP reflects broad CAF activation and is linked to tumor growth, while PDGFR is a more specific indicator of myCAF activation. Therefore, a comparative immunohistochemical evaluation of these markers can provide critical insights into the roles of distinct CAF populations in tumor progression.

In this study, both GP and GS regimens tended to decrease the overall distribution of CAFs. Notably, FAP-positive CAF-like cells significantly decreased in the GS group, while PDGFR-positive CAF-like cells significantly decreased in the GP group. This distinct pattern indicates that, in the TME of PDAC, GP preferentially suppresses PDGFR-positive CAF-like cells and reduces fibrosis, while GS mainly targets FAP-positive CAF-like cells and CAF-associated cytokine signaling. Immunohistochemically, the expression of αSMA, which is highly expressed in myCAFs, decreased in both groups, while the expression of IL-6, an inflammation-related cytokine, was significantly decreased in GS but slightly increased in GP. These results suggest that GP treatment may reduce myCAF-like cells and alleviate stromal fibrosis, whereas GS may exert distinct antitumor effects by reducing FAP-positive CAF-like cells and suppressing substrate remodeling and immunosuppression in the TME. Furthermore, the slight increase in IL-6 expression in the GP group may be attributed to the relative enrichment of iCAFs following the reduction of myCAFs. Mechanistically, this divergence may reflect the distinct pharmacological properties of the applied agents. Paclitaxel, a microtubule-stabilizing agent, strongly disrupts cytoskeletal dynamics and cellular tension, which are critical for the mechanotransduction pathways driving myCAF activation and ECM deposition. Conversely, S-1, an oral fluoropyrimidine derivative, primarily targets nucleic acid metabolism. Because iCAFs exhibit highly active secretory phenotypes driven by dense paracrine signaling loops (such as IL-1/JAK/STAT pathways) to continuously produce inflammatory cytokines like IL-6, they may possess a higher metabolic dependency, rendering them more susceptible to the antimetabolite actions of S-1 than the relatively quiescent myCAFs. The density of CAF marker-positive stromal cells was significantly lower in lung metastatic tumors than in primary tumors, with almost no detectable FAP expression and low levels of αSMA and PDGFR. These findings are consistent with the previous reports of poor CAF induction and activation at metastatic sites [10, 25], suggesting a facilitative effect on further tumor progression by metastasis.

This study has some limitations. The use of immunocompromised nude mice in this study precluded a comprehensive assessment of host immune system–stromal interactions. Furthermore, the functional role of FAP- or PDGFR-positive CAFs as CAF subtypes with respect to direct tumor suppression, the creation of a barrier that collapses blood vessels, and the inhibition of the entry of immune cells and anticancer drugs into tumor tissue were not fully explored [26, 27]. Notably, stromal modulation, such as the suppression of PDGFR-positive CAFs by GP, has been reported with dual PDGFRα/β blockade [28], which enhances the efficacy of immune checkpoint inhibitors in fibrotic tumors. Such overlap would imply that agents inducing CAF remodeling mechanisms comparable to GP would be potential candidates for future combination therapy strategies. Equally compelling is the potential of GS-mediated stromal remodeling. Indeed, analogous observations have been reported in clinical settings; for instance, comparisons of paired human biopsy specimens (pre-neoadjuvant chemotherapy versus post-operative resected tissues) have revealed significant alterations in stromal composition and the tumor microenvironment following treatment [2, 31]. These clinical observations support the concept that neoadjuvant therapy can remodel the tumor stroma. However, direct correspondence with the marker-specific stromal changes observed in our organoid model remains to be established. In future studies, we plan to investigate these stromal alterations using human PDAC specimens to validate our findings and clarify their clinical relevance. In an immunocompetent setting, IL-6 functions as a master regulator of immunosuppression, recruiting myeloid-derived suppressor cells (MDSCs) and excluding cytotoxic T cells to maintain PDAC as an immune-desert or "cold" tumor. The profound suppression of IL-6-expressing iCAFs by GS suggests that this regimen could actively reverse the immunosuppressive TME, potentially sensitizing notoriously resistant PDAC to immune checkpoint inhibitors. Thus, alternating or combining these complementary regimens—GP to dismantle the physical desmoplastic barrier, and GS to extinguish the immunosuppressive cytokine network—could provide a rational strategy to overcome therapeutic resistance. Further detailed analyses using methods such as single-cell analysis and spatial transcriptomics are warranted to elucidate these [29, 30].

In conclusion, both GP and GS regimens not only restricted parenchymal tumor growth but also induced distinct TME remodeling modalities. GP preferentially depleted PDGFR-positive CAF-like cells, thus potentially alleviating desmoplastic physical barriers, whereas GS targeted FAP-positive/IL-6-expressing stromal cells, thereby potentially mitigating immunosuppressive and tumor-promoting cytokine signaling. Conventional single-cell-line xenografts notoriously lack a mature desmoplastic stroma, hindering the evaluation of stroma-targeted therapies. In contrast, our 3D organoid-derived xenograft model successfully recapitulated these nuanced clinical drug responses within a fully developed tumor–stromal architecture. These findings highlight the exceptional utility of this humanized organoid model as a robust preclinical screening platform for precisely predicting the effects of novel investigative compounds, particularly next-generation CAF-targeted therapies or immunomodulatory agents, on the human TME prior to clinical trials.

## Data Availability

The data that support the findings of this study are available from the corresponding authors, Keisuke Taniuchi, upon reasonable request.

## Abbreviations

αSMA: α-Smooth muscle actin
CAF: Cancer-associated fibroblast
CK19: Cytokeratin 19
ECM: Extracellular matrix
EMT: Epithelial–mesenchymal transition
FAP: Fibroblast activation protein
GP: Gemcitabine plus paclitaxel
GS: Gemcitabine plus S-1
H&E: Hematoxylin and eosin
HPF: High-power field
HUVEC: Human umbilical vein endothelial cell
iCAF: Inflammatory cancer-associated fibroblast
IL-6: Interleukin-6
MSC: Human mesenchymal stem cell
myCAF: Myofibroblastic cancer-associated fibroblast
PDAC: Pancreatic ductal adenocarcinoma
PDGFR: Platelet-derived growth factor receptor
SEM: Standard error of the mean
TME: Tumor microenvironment
